# Ethical Considerations in Clinical Trials for Disorders of Consciousness

**DOI:** 10.3390/brainsci12020211

**Published:** 2022-02-02

**Authors:** Michael J. Young, Yelena G. Bodien, Brian L. Edlow

**Affiliations:** 1 Center for Neurotechnology and Neurorecovery, Department of Neurology, Harvard Medical School, Massachusetts General Hospital, Boston, MA 02114, USA; ybodien@mgh.harvard.edu (Y.G.B.); bedlow@mgh.harvard.edu (B.L.E.); 2 Department of Physical Medicine and Rehabilitation, Harvard Medical School, Spaulding Rehabilitation Hospital, Charlestown, MA 02114, USA; 3 Athinoula A. Martinos Center for Biomedical Imaging, Harvard Medical School, Massachusetts General Hospital, Charlestown, MA 02114, USA

**Keywords:** coma, consciousness, neuroethics, philosophy, minimally conscious state, clinical trials

## Abstract

As the clinical trial landscape for patients with disorders of consciousness (DoC) expands, consideration of associated ethical challenges and opportunities is of ever-increasing importance. Responsible conduct of research in the vulnerable population of persons with DoC, including those with coma, vegetative state/unresponsive wakefulness syndrome (VS/UWS), minimally conscious state (MCS), covert cortical processing (CCP), and cognitive motor dissociation (CMD), demands proactive deliberation of unique ethical issues that may arise and the adoption of robust protections to safeguard patients, surrogates, and other key stakeholders. Here we identify and critically evaluate four central categories of ethical considerations in clinical trials involving participants with DoC: (1) autonomy, respect for persons and informed consent of individuals with liminal consciousness; (2) balancing unknown benefits and risks, especially considering the epistemological gap between behavior and consciousness that complicates ordinary ascription of subjective states; (3) disclosure to surrogates and clinical teams of investigational results pertaining to consciousness; and (4) justice considerations, including equitable access to clinical trial enrollment across communities and geographies. We outline guiding principles and research opportunities for clinicians, neuroethicists, and researchers engaged in DoC clinical trials to advance ethical study design and deployment in this complex yet crucial area of investigation.

## 1. Introduction

Mounting mechanistic understanding of human consciousness and its manifold disorders has paved the way for the emergence of numerous promising strategies to detect, predict and promote recovery of consciousness following severe brain injury. As the clinical trial landscape for patients with disorders of consciousness (DoC) expands, consideration of associated ethical challenges and opportunities is of ever-increasing importance. Responsible conduct of research and, especially, clinical trials in the vulnerable population of people with DoC, including persons with coma, vegetative state/unresponsive wakefulness syndrome (VS/UWS), minimally conscious state (MCS), covert cortical processing (CCP), and cognitive motor dissociation (CMD), demand proactive consideration of unique ethical issues that may arise and the adoption of robust protections to safeguard participants, surrogates, and other key stakeholders, while fostering a productive and trustworthy research enterprise.

Here we identify and critically evaluate four central categories of ethical considerations in clinical trials involving participants with DoC: (1) autonomy, respect for persons, and consent of individuals with liminal consciousness and lack of decision-making capacity; (2) balancing unknown benefits and risks, especially considering the epistemological gap between behavior and consciousness that complicates ordinary ascription of subjective states; (3) disclosure of investigational results pertaining to consciousness to surrogates and clinical teams, and data management; and (4) justice considerations, including equitable access to clinical trial enrollment across communities and geographies. While these dimensions by no means exhaust the whole ambit of neuroethics in DoC research, they represent salient and distinctive issues identified in our collective experience designing and conducting clinical trials involving patients with DoC, corresponding ethical and philosophical themes discerned through a review of the literature, and normative analysis. We outline guiding principles for clinicians and researchers engaged in DoC clinical trials to advance ethical study design and deployment in this complex yet crucial research area. While the present analysis focuses on ethical considerations in clinical trials for persons with DoC, many of the issues discussed find similar expression in other forms of research involving persons with DoC.

What differentiates clinical trials from other research studies? Clinical trials are defined by the National Institutes of Health (NIH) as a particular kind of research study in which “human subjects are prospectively assigned to one or more interventions (which may include placebo or other control) to evaluate the effects of those interventions on health-related biomedical or behavioral outcomes” [1]. An intervention is defined by the NIH as any “manipulation of the subject or subject’s environment for the purpose of modifying one or more health-related biomedical or behavioral processes and/or endpoints” [2]. The interventional nature of clinical trials heightens the need for rigorous ethical safeguards to ensure that participants are responsibly enrolled, their welfare prioritized, and protected against inappropriate risks [3,4,5,6]. This is especially the case for clinical trials involving participants with DoC whose ability to actively communicate with investigators is constitutively dampened by their brain state. As of 16 December 2021, over 200 active or pending clinical trials involving subjects with DoC were registered on ClinicalTrials.Gov; these include both observational and interventional trials involving devices, drugs, stimulation techniques, and diagnostic procedures. Each of these interventions carries a unique balance of benefits and risks, and it is beyond the present scope to extensively catalog the normative features of each investigational approach. Rather, our aim is to elucidate key shared ethical dimensions of DoC clinical trials and to evaluate their overarching implications for clinicians, investigators, patients, and surrogates.

## 2. Preserving Patient Autonomy in Clinical Trials Involving Persons with DoC

“Autonomy” etymologically derives from the Greek roots “autos” (“self”) + “nomos” (“law”) [7,8,9,10]. Harboring and exercising autonomy entails the faculty and practice of living one’s life in accordance with one’s own choices, values, and goals; the autonomous individual is thus self-governing, insofar as the laws, broadly construed, dictating the course of key decisions are self-derived and concordant with one’s own vital aims. Accordingly, respecting patient autonomy in the context of clinical practice and research requires integrating patient preferences and values in decisions surrounding treatment pathways, goals of care, determination of best-interests, and participation in research studies and clinical trials. The process of voluntary and informed consent is anchored to preserving patient autonomy; in its absence, the risk of decisions being made that contravene patient preferences and thus abrogate a patient’s self-rule is acutely heightened [11,12,13,14]. The doctrine of informed and voluntary consent in clinical medicine and research was ratified in the Nuremberg Code, Belmont Report and Declaration of Helsinki, and later crystalized in canonical work by bioethicists and moral philosophers, including Beauchamp and Childress’ Principles of Biomedical Ethics [15,16,17,18,19,20].

Recognizing the potential for conflicting obligations that might be experienced by clinicians/investigators tasked with balancing professional duties deriving from the doctor–patient relationship with, at times, competing duties toward society and science writ large, the Declaration of Helsinki, adopted by the World Medical Association in 1964 and updated in 2013, emphasizes that clinicians/investigators should never prioritize the pursuit of scientific knowledge over the welfare of the individual research subject. “While the primary purpose of medical research is to generate new knowledge”, the Declaration stresses, “this goal can never take precedence over the rights and interests of individual research subjects. It is the duty of physicians who are involved in medical research to protect the life, health, dignity, integrity, right to self-determination, privacy, and confidentiality of personal information of research subjects” [21]. The Declaration further specifies that, when a physician discusses a research study with a patient or surrogate, he or she “must fully inform the patient which aspects of their care are related to the research. The refusal of a patient to participate in a study or the patient’s decision to withdraw from the study must never adversely affect the patient–physician relationship” [21].

Regulatory codes and conventions in the United States and European Union recapitulate these ethical imperatives. In the United States, requirements for obtaining informed consent from research subjects are codified in the Code of Federal Regulations (CFR), Title 21, Chapter 1, Part 50, “Informed Consent of Human Subjects” [22]. The relevant portion of CFR specifies that “no investigator may involve a human being as a subject in research covered by these regulations unless the investigator has obtained the legally effective informed consent of the subject or the subject’s legally authorized representative. An investigator shall seek such consent only under circumstances that provide the prospective subject or the representative sufficient opportunity to consider whether or not to participate and that minimize the possibility of coercion or undue influence. The information that is given to the subject or the representative shall be in language understandable to the subject or the representative. No informed consent, whether oral or written, may include any exculpatory language through which the subject or the representative is made to waive or appear to waive any of the subject’s legal rights, or releases or appears to release the investigator, the sponsor, the institution, or its agents from liability for negligence” [22]. The CFR also details exceptions to the general requirements of informed consent, including situations in which the “human subject is confronted by a life-threatening situation necessitating the use of the test article, informed consent cannot be obtained from the subject because of an inability to communicate with, or obtain legally effective consent from, the subject, time is not sufficient to obtain consent from the subject’s legal representative, and there is available no alternative method of approved or generally recognized therapy that provides an equal or greater likelihood of saving the life of the subject” [22]. Who qualifies as a “legally authorized representative” to provide proxy consent for research participation varies by jurisdiction [23]. While our focus here is primarily on ethical frameworks within United States and European Union, and it is beyond the present scope to canvas the complexities of consent across all regions and jurisdictions, it is important to note that approaches to informed consent in and across other regions are highly varied. Lacunae in regulatory standards of informed consent and research ethics in some regions underscore a significant need for global harmonization of approaches to informed consent to protect research participants [24,25,26,27,28,29,30]. Less developed regulatory oversight in some regions has contributed to a trend of “outsourcing” of global clinical trials to jurisdictions where clinical-trial conduct is perceived to be less burdensome, including areas of Eastern Europe, China, India South America, and elsewhere [26,31,32,33,34,35,36]. While some sponsors might be drawn to such practices, which some have described as a “strategic imperative” [37], it is vital to recognize that possibilities for vulnerable research participants to be exploited or unduly harmed are magnified if sufficiently robust ethical protections are lacking [36,38]. Opportunities to align global research ethics through supranational regulatory machinery and international cooperation should be recognized and strengthened. Divergent philosophical perspectives and attitudes surrounding autonomy, paternalism, and the role of the individual in society have inspired varied approaches to conceptualizing and prioritizing informed consent across cultures. For example, collectivist conceptions of personhood, family, and interdependency have been theorized to influence distinctively family-oriented paradigms of informed consent in China and Japan [39,40,41,42,43,44]. The need to advance the rigor and integrity of informed-consent procedures across regions represents a challenge and opportunity; building cross-cultural bridges to study and integrate divergent philosophies of autonomy with the common aim of protecting the safety and well-being of research participants, and particularly those who may lack decisional capacity, such as persons with disorders of consciousness, is a growing ethical imperative [45,46,47,48,49].

In 2019, a revised US Common Rule came into effect that, inter alia, requires that “[f]or each clinical trial conducted or supported by a Federal department or agency, one IRB approved informed consent form used to enroll subjects must be posted by the awardee or the Federal department or agency component conducting the trial on a publicly available Federal Web site that will be established as a repository for such informed consent forms …. no later than 60 days after the last study visit by any subject, as required by the protocol”. When explaining the rationale for this new requirement, it is specified that the “primary purpose of this provision is to improve the quality of consent forms in federally funded research by assuring that—contrary to current practices, under which it is often very difficult to ever obtain a copy of these documents—they eventually would become subject to public scrutiny and that they will provide useful models for others. The consent form plays a key role in making sure that someone asked to enter a clinical trial receives the information they be making decision about whether to enroll in that trial. Accordingly, it also plays a key role in supporting and justifying the public’s trust in the integrity of our clinical trial enterprise… Fundamentally, this proposal is about increasing the transparency of one of the most important aspects of our human subjects protection system. Increased transparency is in general a good thing, and in this instance, as in many others, it offers multiple benefits—including increased trust—at very low cost”. Compliance with this requirement has been variable across study sites and types [50].

In European Union (EU) member states, legal and regulatory standards around informed consent stem from the Convention on Human Rights and Biomedicine (Oveido Convention), which has been in force since 1999, and EU Regulation No. 536/2014 (“clinical trials on medicinal products for human use”), which replaces the precedent Directive 2001/20/EC [51,52,53]. The General Data Protection Regulation 2016/679 (GDPR) provides additional guidance and protections around data use, consent, processing, and privacy, with some variation in interpretation by state [54,55,56,57,58]. The Oveido Convention (Article 5—General Rule) details that “[a]n intervention in the health field may only be carried out after the person concerned has given free and informed consent to it. This person shall beforehand be given appropriate information as to the purpose and nature of the intervention as well as on its consequences and risks” [59]. Article 6 (“Protection of Persons not able to Consent”) includes exceptions to the general rule for those who cannot consent, explaining that “[w]here, according to law, an adult does not have the capacity to consent to an intervention because of a mental disability, a disease or for similar reasons, the intervention may only be carried out with the authorisation of his or her representative or an authority or a person or body provided for by law. The individual concerned shall as far as possible take part in the authorisation procedure” [59]. Article 16 (“Protection of Persons undergoing Research”) stipulates the following general requirements:

“Research on a person may only be undertaken if all the following conditions are met: (i) there is no alternative of comparable effectiveness to research on humans; (ii) the risks which may be incurred by that person are not disproportionate to the potential benefits of the research; (iii) the research project has been approved by the competent body after independent examination of its scientific merit, including assessment of the importance of the aim of the research, and multidisciplinary review of its ethical acceptability; (iv) the persons undergoing research have been informed of their rights and the safeguards prescribed by law for their protection; (v) the necessary consent as provided for under Article 5 has been given expressly, specifically and is documented. Such consent may be freely withdrawn at any time” [59].

Article 17 (“Protection of persons not able to consent to research”) adds further requirements for conducting research involving persons who cannot consent, specifying that “[r]esearch on a person without the capacity to consent as stipulated in Article 5 may be undertaken only if all the following conditions are met: (i) the conditions laid down in Article 16, sub-paragraphs I to iv, are fulfilled; (ii) the results of the research have the potential to produce real and direct benefit to his or her health; (iii) research of comparable effectiveness cannot be carried out on individuals capable of giving consent; (iv) the necessary authorisation provided for under Article 6 has been given specifically and in writing; and (v) the person concerned does not object” [59].

For research that does not have the potential to produce direct benefits to the health of the participant who cannot consent (i.e., that fails to meet criterion ii), Article 17 adds the following exceptional condition:

“… where the research has not the potential to produce results of direct benefit to the health of the person concerned, such research may be authorised subject to the conditions laid down in … i, iii, iv and v above, and to the following additional conditions: (i) the research has the aim of contributing, through significant improvement in the scientific understanding of the individual’s condition, disease or disorder, to the ultimate attainment of results capable of conferring benefit to the person concerned or to other persons in the same age category or afflicted with the same disease or disorder or having the same condition; (ii) the research entails only minimal risk and minimal burden for the individual concerned” [59].

EU Regulation No. 536/2014 specifies additional conditions and requirements for performing research involving subjects who are not able to consent [60].

These requirements are typically operationalized by institutional internal review boards (IRBs) and may be impacted by other local rules and regulations [61]. Challenges posed by the COVID-19 pandemic motivated changes to the informed-consent process in many settings, with increased reliance on electronic consent, waivers, or remote consent methods, due to heightened infection risk posed by face-to-face informed-consent conversations [62,63,64].

Clinical trials involving participants with DoC pose a distinct challenge to the doctrine of informed consent insofar as participants by definition are unable to actively engage in decision-making [65]. However, discovery of novel therapies for this population is crucial, with survivors facing severely limited treatment options and high rates of mortality, morbidity, and long-term neurological disability [66,67,68,69,70]. Recognizing this challenge, Fins and colleagues incisively state that “[p]aradoxically… those with disorders of consciousness often have been protected from the research enterprise by regulatory exclusion, which inverts the justice ethos of Belmont” [71]. Instead, they argue that there is a “need for inclusion of decisionally incapacitated subjects in research as long as it is ethically proportionate and attentive to foreseeable risks and benefits… The ethical challenge for investigators is to study this population to learn more about their cognitive capabilities and to include these subjects, as they are able, in the consent or assent process, recognizing that subjects may reveal a greater capability to engage with the investigative team” [71] as a result of the research being conducted or in the natural history of recovery. Thus, rather than categorically disenfranchising incapacitated persons with DoC from clinical trials due to inability to provide direct consent, alternative models to responsibly study persons with DoC have been adopted by investigators. These models include surrogate (proxy) consent, consensus consent, deferred consent, community consultation, and waiver of consent [72,73,74,75,76,77,78]. Each of these models endeavors to uphold participant autonomy and protect patient welfare, despite the inability to obtain direct, synchronous informed consent from the person with DoC.

In other contexts involving acutely injured or critically ill patients, researchers and clinicians have worked closely with ethicists to design study paradigms balancing principles of respect for patient autonomy, beneficence, justice, and non-maleficence, whilst facilitating research inclusion. Alternative paradigms of consent that may ensure protection of subjects’ autonomy without restricting responsible participation in research include the following: (i) surrogate consent (by legally appointed or default proxy decision-maker), (ii) waiver of informed consent utilizing a federal exception from informed consent (EFIC), (iii) deferral of consent with retrospective debriefing, (iv) a consent substitute model, and (v) community consultation [79,80,81,82,83,84,85]. Elements of each of these paradigms might be variably integrated into a hierarchical, adaptive, or multimodal system of informed consent to be deployed in a DoC clinical trial where variation in participant capacity or surrogate availability is anticipated.

Independently, each of these models carries a distinctive array of ethical and operational benefits and challenges. For example, while surrogate consent is perhaps the most ubiquitous approach when conducting research involving incapacitated persons, the ability of a surrogate to provide consent may be precluded by restricted time windows to enrollment, or may be compromised by psychological stress induced by a high-acuity brain injury in a loved one. Moreover, surrogate decision-makers are often unknown or unavailable in acute settings, and where they are available, they may not be familiar with the participant’s wishes [86,87,88,89,90,91,92,93].

In 1996, recognizing the growing need to include patients with emergent pathologies in research despite a lack of capacity commonly occasioned by high-acuity and time-sensitivity, the United States Food and Drug Administration (FDA) and Department of Health and Human Services (HHS) developed regulations that allowed for studies to proceed in acute situations where current treatment paradigms are considered unsatisfactory. These are known as EFIC regulations and waiver of informed consent (WIC) regulations [94]. Whereas EFIC regulations apply to research involving drugs or devices, WIC regulations apply to research involving non-FDA regulated modalities, such as comparisons of standards of care. Over the past 25 years, the FDA has granted over 40 EFICs for trials that have collectively enrolled over 40,000 patients, including trials of interventions and devices for cardiac arrest, septic shock, traumatic brain injury, status epilepticus, stroke, respiratory failure, and acute coronary syndrome [82,95]. However, the processes required to satisfy these requirements are rigorous and demand meticulous attention to ensure ethical appropriateness and regulatory adherence [94,96]. Deferral of consent procedures, whereby patients are asked to provide informed consent only after enrollment or potentially recovery, have occasionally been used in emergency research, including in several large trials of acute ischemic stroke interventions, but subsequent analyses have questioned these methods [97]. Learning from these results, some trialists have crafted shorter consent processes as a more practical approach to achieving research aims while preserving patient autonomy [98,99]. However, merely shortening the consent process does not solve the challenges of patient incapacity or surrogate unavailability. Advocates of consent substitute models contend that formal informed consent can be bypassed if core normative demands underpinning the informed consent process are met. One such model proposes that consent may be substituted if an investigational intervention (a) treats a medically urgent need in incapacitated patient; (2) has a comparable risk–benefit ratio to standard of care; (3) does not pose discernible conflict with patient values; (4) carries minimal net risk; and (5) ensures that, when it becomes possible, informed consent for continued research is obtained from patients or surrogates [79,100]. Fins and colleagues describe an approach of “consensus consent” when considering enrolling persons with DoC in clinical trials, which requires involvement and agreement among four parties—the participant’s “legally authorized representative, the subject’s physician, the clinical investigator, and a lay subject advocate” [101,102,103]. Others contend that a model of interactive community consultation or public deliberation, whereby salient values and preferences of recovered patients and other key stakeholder groups are ascertained in advance of a planned study, could potentially supplant or supplement the traditional informed consent process in select circumstances [81,104,105,106,107]. Despite the proliferation of alternative models to informed consent, ethical debate still exists regarding the optimal form these approaches should take.

As alternative models of consent continue to mature, a precautionary approach that is maximally responsive to the standards articulated by the WMA Declaration of Helsinki and local IRB is indicated. Articles 28–30 of the revised (2013) WMA Declaration of Helsinki emphasize the following:

“For a potential research subject who is incapable of giving informed consent, the physician must seek informed consent from the legally authorised representative. These individuals must not be included in a research study that has no likelihood of benefit for them unless it is intended to promote the health of the group represented by the potential subject, the research cannot instead be performed with persons capable of providing informed consent, and the research entails only minimal risk and minimal burden…When a potential research subject who is deemed incapable of giving informed consent is able to give assent to decisions about participation in research, the physician must seek that assent in addition to the consent of the legally authorised representative. The potential subject’s dissent should be respected…Research involving subjects who are physically or mentally incapable of giving consent, for example, unconscious patients, may be done only if the physical or mental condition that prevents giving informed consent is a necessary characteristic of the research group. In such circumstances the physician must seek informed consent from the legally authorised representative. If no such representative is available and if the research cannot be delayed, the study may proceed without informed consent provided that the specific reasons for involving subjects with a condition that renders them unable to give informed consent have been stated in the research protocol and the study has been approved by a research ethics committee. Consent to remain in the research must be obtained as soon as possible from the subject or a legally authorised representative” [21].

This approach combines elements of surrogate consent with deferred consent/assent and importantly requires that unconscious patients only be studied if the condition that renders them decisionally incapacitated is a necessary feature of the research being conducted; so, for example, it would not be considered ethically acceptable to enroll a hospitalized patient who is comatose due to TBI in a clinical trial studying a novel antimicrobial for treatment of pneumonia (even if that patient is affected by pneumonia), as the condition that prevents them from personally consenting (TBI) is not a necessary feature of the research group.

A 2020 American Academy of Neurology (AAN) position statement on ethical issues in clinical research in neurology, authored by the Ethics, Law, and Humanities joint committee of the AAN, American Neurological Association, and Child Neurology Society, articulates similar standards for informed consent in situations where participants’ decision-making capacity is impaired [108]:

“When impaired decision-making capacity precludes direct fulfillment of the requirement for informed consent, a legally authorized representative may make the decision on the participant’s behalf. The 2017 amendment to the Common Rule specifies that, where state law does not specify rules for surrogate decision-making about research, surrogates for medical decision-making can also be considered surrogates for decisions about clinical research (this is the case in most states). The surrogate should use a substituted judgment standard, making the decision based on the participant’s historical beliefs and values, as the surrogate believes the participant would, were the participant able to undertake the informed consent process. IRBs may require additional safeguards, such as independent consent monitors, depending on the degree of cognitive incapacity and the study’s risk–benefit ratio and complexity. In developing such safeguards, IRBs should make every effort to fully protect participants with dementia and other cognitive impairments, while minimizing impediments to studies of these important conditions. Similarly, in developing safeguards for pediatric participants, IRBs should endeavor to balance rigorous protection for children participating in research, with the minimization of impediments to clinical research on pediatric neurologic disorders. Of note, the informed consent process can be cognitively and emotionally demanding, and every effort should be made to ensure participants’ and surrogate decision-makers’ comprehension. In addition, where possible, researchers should consider disclosing relevant conflicts of interest during the informed consent process” [108].

When involving surrogate decision-makers in discussions surrounding substituted consent for trial participation, it is crucial for investigators to avoid language that might lead to therapeutic misconception or false expectations, to pressure surrogates to enroll, or to suggest that the investigational intervention has established clinical benefit [109,110,111,112,113]. To safeguard against these risks, some investigators first approach the prospective participant’s primary medical team about the surrogate’s readiness to discuss research opportunities, and only if there is agreement will a trained clinical research coordinator approach the surrogate decision maker in a non-pressured environment with the opportunity to ask any questions and voice any concerns [77]. To ensure adequate comprehension, plain language and interactive informed-consent methods may be adopted incorporating multimedia (e.g., introductory video), interactive questionnaires, and other novel techniques [114,115,116,117,118,119]. The informed-consent process should ideally clarify, in plain language, why the clinical trial is being performed, who might benefit from the results, how long the trial will last, what will happen if one chooses to take part in the study, what will happen after the study ends, why one might choose not to participate in the study, what other interventions are available, who to call with questions or concerns about the study, possible benefits and risks, what will happen if one wants to drop out after enrolling, whether reimbursement will be offered for participation, what information will be collected through the study, how privacy will be protected, and how results will be disseminated.

Consent ought to be viewed as an ongoing dialectical process, rather than as an episodic requirement [120,121,122]. If in the course of a trial a participant with DoC experiences recovery (or discovery) of functional communication, investigators should consider approaching the participant to provide information about the study in which they are enrolled, and offer the participant the opportunity to affirm their consent, assent, or dissent, and this decision should be respected. For participants who are found to be covertly conscious—that is, those who appear behaviorally unresponsive to simple commands but demonstrate the ability to volitionally modulate brain activity under active neuroimaging or EEG paradigms—important unanswered questions exist about whether and how investigators should approach obtaining deferred consent. Empirical neuroethics research is needed to better understand the attitudes of patients and families surrounding the optimal approach to involving research participants with emerging consciousness in the consent process, recognizing the pitfalls of assigning too much or too little weight to surrogate indicators of subjective attitudes ascertained through processed neural correlates. Efforts to restore functional communication through brain–computer interfaces and other assistive technologies in persons with covert consciousness are ongoing, and, in the future, they may aid in safeguarding the autonomy and welfare of these especially vulnerable persons and improve the ethical resilience of longitudinal DoC research programs [123,124,125,126,127,128].

## 3. Balancing Benefits and Risks in DoC Clinical Trials

Preserving participant autonomy through substituted consent is a necessary but not sufficient condition to ensure ethical DoC clinical trial design and deployment. Drawing upon elements from dominant threads of moral philosophy and codes of ethics, Emmanuel and colleagues detail six other ethical requirements in evaluating clinical research: [129].

(1) “Value—enhancements of health or knowledge must be derived from the research”;

(2) “Scientific validity—the research must be methodologically rigorous”;

(3) “Fair subject selection—scientific objectives, not vulnerability or privilege, and the potential for and distribution of risks and benefits, should determine communities selected as study sites and the inclusion criteria for individual subjects”;

(4) “Favorable risk–benefit ratio—within the context of standard clinical practice and the research protocol, risks must be minimized, potential benefits enhanced, and the potential benefits to individuals and knowledge gained for society must outweigh the risks”;

(5) “Independent review—unaffiliated individuals must review the research and approve, amend, or terminate it”;

(6) “Respect for enrolled subjects—subjects should have their privacy protected, the opportunity to withdraw, and their well-being monitored” [129].

Of these elements, determination of the risk–benefit ratio in DoC clinical trials may prove particularly challenging, as participants with DoC typically cannot report on their subjective experiences following an intervention. Risks may thus be unknown or imperceptible, and benefits could be difficult to rigorously appraise. While behavior-based, electrophysiologic, or neuroimaging indices of improvement might be measured, the epistemological gaps between behavior, brain activity, and subjective phenomenology complicate ordinary ascription of subjective states on the sole basis of an observed change in behavior or brain activity. Complicating matters further, instruments intended to measure the broad range of possible outcomes after severe brain injury are coarse and often dichotomized in “favorable” and “unfavorable”, categories that are arbitrarily defined by investigators and clinicians rather than by patients and caregivers [130]. Moreover, normative preferences surrounding intensity of treatment in the event of DoC are often unknown, and quality of life within DoC may be difficult to ascertain and track. This lack of uniformity surrounding what constitutes a favorable outcome in persons with DoC complicates the process of determining which factors should qualify as a relevant benefit, and the relative weight of each factor. The imperviousness of subjective psychological experiences of those with DoC could thus lead to skewed risk–benefit assessments when evaluating the promises and pitfalls of novel interventions [131,132,133].

To improve the understanding and communication of risks and benefits associated with DoC clinical trial participation, investigators should disambiguate direct, indirect, absolute, and relative risks and potential benefits, and when possible, endeavor to estimate the magnitude and likelihood of each [134]. This process has been referred to as component analysis [135,136]. Consider the example of a clinical trial investigating the efficacy of a novel drug to promote recovery of consciousness in patients with coma, and which utilizes fMRI signatures of consciousness as a pharmacodynamic biomarker of response (clinicaltrials.gov NCT03814356) [137]. Direct risks might include those posed by the drug itself (e.g., side effects of study drug). Indirect risks might include idiosyncratic interactions with other medications or indirect consequences of prolonged recumbency in the MRI scanner. Potential direct benefits might include the possibility, albeit not guaranteed, that the study drug may help restore consciousness faster than the pace of natural recovery, even if it is made clear that the study is not designed to restore consciousness but rather to understand safety and pharmacokinetics. Indirect potential benefits might include the possibility that information collected from the study may help researchers find novel future treatments, or increased interaction time with clinicians/investigators. The possibility that unknown psychological effects may occur with interventions in persons with DoC should be explained transparently, and clinical equipoise should be confirmed (defined as “a state of genuine uncertainty on the part of the clinical investigator regarding the comparative therapeutic merits of each arm” [138,139]). After distinguishing and cataloging potential benefits and risks entailed by a given clinical trial design, investigators should consider possible modifications to the study design to mitigate risks and maximize benefits, evaluate the cumulative balance of foreseeable benefits and risks (both to the subject and society), and determine, with the aid of a local IRB, whether the anticipated cumulative balance of risks and benefits is proportionate and reasonable [134,140,141,142,143,144].

Given the gaps of knowledge that exist in this area, growing opportunities exist for clinicians/investigators to collaborate with neuroethicists to empirically study the nature of benefits and risks of novel DoC interventions, as informed by perspectives of families and other key stakeholders involved in clinical trials [145,146].

## 4. Handling Investigational Results Pertaining to Consciousness

The determination of whether, when, and how to disclose research results requires case-by-case consideration, given the plurality of factors that might bear on these decisions and their potential impact on participants, surrogates, and research-data integrity. These questions should be proactively discussed during the study design phase and written into the protocol and consent procedure [147,148,149,150,151]. Consensus has been growing among ethicists, legal scholars, and regulators in favor of more regular return of research results, where appropriate [152], and a 2018 Consensus Study Report of the National Academies of Sciences, Engineering, and Medicine (NASEM) details a vision for “Returning Individual Research Results to Participants: Guidance for a New Research Paradigm” [149]. The NASEM report was commissioned by the Centers for Medicare and Medicaid Services (CMS), the Food and Drug Administration (FDA), and the National Institutes of Health (NIH). The NASEM framework emphasizes that “[a]s the potential value of the result to participants and the feasibility of return increase, the justification for returning results becomes stronger” [149]. Value need not be defined in strictly clinical terms. The NASEM report expounds the following:

“Value in this context means the value of a result from the perspective of the participant and might entail clinical utility or personal utility as well as personal meaning (e.g., lineage information). This participant-centric approach recognizes that the value of a result is not necessarily tied to its use. To clarify, defining value in this way is not meant to imply that each participant needs to be queried regarding the results that would be meaningful to him or her, but it does require the investigator to consider value from the participant perspective rather than from the more traditional clinical perspective. Feasibility is also determined by multiple factors, including potential challenges, the costs and burdens of returning results, whether biospecimens can be linked to a specific participant, and the resources available to communicate the results effectively and appropriately” [149].

Similarly, the HHS Secretary’s Advisory Committee on Human Research Protections (SACHRP) has clarified that “there should be due consideration of whether to return individual research results during the design, conduct, and oversight of research, with a general but rebuttable presumption towards disclosure” [153]. In describing the ethical basis for this guidance, SACHRP specifies the following:

“Provision of individual research results to research subjects is supported by the principles of Respect for Persons and Beneficence. Subjects make an autonomous decision to participate in research, and in so doing help to create scientific knowledge that is valuable to society and to other individuals. One means to provide recognition and appreciation for this contribution is to provide subjects with their individual information that results from the research. This can help to build a sense of participation and partnership in the development, and makes their experience more fulfilling. For these reasons, this policy has the potential to lead to greater public trust in research and greater willingness by individuals to participate in research, which will increase the development of new knowledge and interventions for the public good. In addition, the information may (if sufficiently reliable and valid) prove valuable for the subjects or their families in making future decisions regarding their health or welfare, such as future choices about the use of certain classes of drugs or family planning decisions…the individual results do not have to be of clinical value to the subjects in order for return to be considered. Even if the results are not clinically relevant, the pure intellectual curiosity of the subjects is sufficient reason to return the results absent other reasons not to return them … SACHRP believes it is often appropriate as an ethical issue to return individual results to research subjects, with a stronger presumption as the validity and actionabililty increase” [153].

Similarly, the Oveido Convention emphasizes that “[e]veryone is entitled to know any information collected about his or her health. However, the wishes of individuals not to be so informed shall be observed” (Article 10) [59]. The salient ethical threads of reciprocity, transparency, beneficence, and autonomy that run through these positions, thus, generally support the return of research results to patients or surrogates. While the forgoing positions do not specifically address how these considerations might be affected in contexts where participants are unable to directly consent to participate in research, such as in clinical trials involving patients with DoC, it may reasonably follow from the principles discussed that the propriety of returning research results is amplified in contexts of research participant incapacity.

During a clinical trial or research study involving participants with DoC, it is possible that results will be obtained that shed light on the participant’s level of consciousness, including findings suggestive of covert consciousness, or CMD, wherein the level of awareness detected on fMRI and EEG exceeds that which is observed on bedside neurobehavioral examination. In other circumstances, investigational results might challenge the likelihood of conscious awareness or recovery thereof in clinical contexts of diagnostic and prognostic uncertainty. In such circumstances, important questions arise surrounding whether, how, and to whom such investigational results ought to be disclosed, and how to characterize the value of such information [70,146,154,155,156,157].

In contrast to typical “incidental findings”, results such as these may be anticipated, intentionally sought after (e.g., to aid a clinical team in neuroprognostication), or generated by design [158,159,160,161]. Insofar as such results may inform surrogates’ and clinical teams’ judgements about the presence or absence of consciousness and possibility of recovery, there is a strong ethical case to be made for routine disclosure of investigational results pertaining to consciousness, with appropriate counseling and provision of anticipatory guidance, especially if such information might bear on therapeutic approaches and goals of care conversations. Withholding such information from clinicians or family members, even if the information is investigational in nature and non-conclusive, may be viewed as running contrary to the ethical principles of autonomy, transparency, reciprocity, and beneficence [162,163,164,165,166]. Arguments in favor of non-disclosure may appeal to concerns of affecting the integrity of the research study or study outcomes (e.g., if un-blinding occurs or if the results influence subsequent longitudinal data acquisition), confusing family members or clinicians, reinforcing false hope (in the case of an unduly optimistic study result), or generating false despair (in the case of a unduly pessimistic study result) [167,168,169]. To manage these concerns and mitigate risks, investigators should work closely with ethicists to develop a standardized approach to sensitively handle investigational results pertaining to the presence or absence of consciousness. Qualitative research findings by Peterson and colleagues have challenged concerns surrounding therapeutic misconception and false hope, suggesting that caregivers generally understood results of advanced neuroimaging pertaining to consciousness and that “pre-disclosure consultations” and standardized methods of disclosure could improve reception and understanding of complex results [156]. To align expectations, surrogate decision-makers should be proactively counseled about the nature of the information that might accrue through study participation and their attitudes toward receiving or not receiving such information gauged. A harmonized process could accordingly be developed, analogous to AAN ethics committee guidance on handling of neurogenetic research findings “with the ultimate objective of providing participants with an option to be informed—or not—of incidentally discovered actionable results during the informed consent process prior to” testing [108]. Investigators should sensitively discuss all clinically relevant findings with the primary medical team, and they should work collaboratively to devise a responsible approach to disclosing results to surrogates in a manner that acknowledges the inherent ambiguities that might be latent in the investigational data and its defeasible interpretation [154,170,171,172,173].

As the acquisition of such patient data becomes more common with the guideline-driven clinical translation of advanced neurotechnologies to detect and predict recovery of consciousness [174,175], these ethical quandaries will undoubtedly evolve and multiply. Nascent opportunities, therefore, exist for embedded neuroethics research to capture and critically evaluate the experiences and perspectives of key stakeholders involved in such studies, particularly surrounding information disclosure practices. Investigators must work closely with clinicians, IRBs, and ethicists to harmonize approaches to disclosing such findings to primary medical teams and families, informed by emerging neuroethics principles and research [146].

## 5. Advancing Equity in DoC Clinical Trial Access and Enrollment

Despite an increase in DoC clinical trials over the past decade, access and enrollment remains highly irregular, especially in remote or underserved areas. Racial-, ethnic-, and language-based disparities are also prevalent across a variety of clinical trial domains [176,177,178,179,180,181]. Ethical considerations of justice and equity demand close attention to these potential disparities and necessitate strategies to democratize access to DoC clinical-trial enrollment [67,69,70]. Research identifying patterns of accrual of participants in DoC clinical trials, characterizing disparities, and illuminating barriers to enrollment is needed to ensure diverse and equitable clinical-trial enrollment. Strategies to enhance access to DoC clinical trials in underserved and remote areas are needed. Hub-and-spoke model systems that rely on networks of central tertiary DoC research hubs with connections to community medical centers may provide a platform for expediently triaging patients in need of specialty DoC management or who might be interested in clinical trial enrollment [154,182,183]. Nonetheless, many logistical challenges exist, including financial complexities, transportation across state or country borders, and regulatory barriers. While these challenges have received substantial attention in other fields of research, such as clinical trials for cancer and other disease areas [184,185,186,187,188,189,190,191,192,193,194,195,196,197], their role in DoC research studies has been relatively underexplored and represents a vital area for ongoing advocacy and investigation. Initiatives to enhance equity in clinical-trial enrollment, including resources to promote diverse and inclusive enrollment and training for researchers, have been made available by FDA [198]. The NIH Revitalization Act of 1993, the 21st Century Cures Act (2016), NIH Policy and Guidelines on the Inclusion of Women and Minorities as Subjects in Clinical Research (2017), and the FDA guidance on “Enhancing the Diversity of Clinical Trial Populations—Eligibility Criteria, Enrollment Practices, and Trial Designs Guidance for Industry” (FDA-2019-D-1264) outline additional steps to advance diverse and equitable representation in clinical trials.

## 6. Ethically Informed DoC Clinical Trial Design and Execution

Clinical trials for DoC promise to fill fundamental gaps in intervention and prognostication for some of the most vulnerable and marginalized patients [199,200]. Such trials, therefore, present tremendous opportunities, while also raising challenging ethical and social issues. While canvasing every ethical issue pertaining to each type of intervention is well beyond the scope of the present analysis, the framework developed here emphasizes four important shared ethical dimensions of DoC clinical trials that ought to be considered by investigators when designing and executing trials: (1) autonomy and informed consent for persons with liminal consciousness; (2) balancing and communicating atypical and often unknown benefits and risks; (3) disclosure of investigational results pertaining to consciousness to surrogates and clinical teams; and (4) justice considerations, including equitable DoC clinical trial access and accrual (Table 1). Ethics engagement should not be viewed as a regulatory barrier or impediment to research progress, but rather as an opportunity to promote the long-term success, trustworthiness, and resilience of DoC clinical trials and results. There are also emerging funding opportunities to support embedded neuroethics research and scholarship, sponsored by initiatives such as NIH BRAIN [201]. Recognizing that a “one size fits all” approach will likely overlook important normative nuances and needs of each individual trial, investigators should proactively partner with neuroethicists and local IRBs in ensuring ethically informed DoC clinical trial design and execution.

## Figures and Tables

**Table 1:** DoC Clinical Trials: Salient Ethical Challenges, Considerations for Researchers & Selected Regulatory Guidance.

Ethical Challenge	Key Considerations for Researchers	Relevant Regulatory Codes & Conventions
**Autonomy and informed consent for persons with liminal consciousness**	Voluntary and informed consent is anchored to respect for autonomy. Instead of disenfranchising patients with DoC from research participation due to decisional incapacity, trials involving participants with DoC who cannot consent must consider alternative pathways to ensure responsible inclusivity and preserve participant autonomy, including surrogate consent.	Convention on Human Rights and Biomedicine (Oveido Convention) EU regulation No 536/2014 (“clinical trials on medicinal products for human use”); Directive 2001/20/EC Code of Federal Regulations (CFR); Common Rule Declaration of Helsinki (1964, updated 2013) Belmont Report
**Balancing and communicating atypical and often unknown benefits and risks**	Determination of risks and benefits in DoC clinical trials may prove particularly challenging, as participants with DoC typically cannot report on their subjective experiences. Risks may thus be unknown or imperceptible, and benefits could be difficult to rigorously appraise. While behavior-based, electrophysiologic, or neuroimaging indices of improvement might be measured, the epistemological gaps between behavior, brain activity and subjective phenomenology complicate ordinary ascription of subjective states on the sole basis of an observed change in behavior or brain activity. To improve the understanding and communication of risks and benefits associated with DoC clinical trial participation, investigators should disambiguate direct, indirect, absolute and relative risks and potential benefits, and when possible, endeavor to estimate the magnitude and likelihood of each [134,135,136].	Declaration of Helsinki (1964, updated 2013) Belmont Report Code of Federal Regulations NIMH Guidance on Risk-Based Monitoring
**Disclosure of investigational results pertaining to consciousness to surrogates and clinical teams**	During a clinical trial involving participants with DoC, it is possible that results will be obtained that shed light on the participant’s level of consciousness and/or capacity for recovery in clinical contexts of diagnostic and prognostic uncertainty. In such circumstances, important questions arise surrounding whether, how and to whom such investigational results ought to be disclosed, and how to characterize the value of such information [70,146,154,155,156,157]. Determination of whether, when and how to disclose research results demands case-by-case consideration, given the plurality of factors that might bear on these decisions and their potential impact on participants, surrogates and research data integrity. These questions should be proactively discussed during the study design phase and written into the protocol and consent procedure [147,148,149,150,151]. Consensus has been growing among ethicists, legal scholars and regulators in favor of more regular return of research results where appropriate. Opportunities exist for embedded neuroethics research to capture and critically evaluate the experiences and perspectives of key stakeholders involved in such studies, particularly surrounding information disclosure practices. Investigators must work closely with clinicians, IRBs and ethicists to harmonize approaches to disclosing such findings to primary medical teams and families, informed by emerging neuroethics principles [146].	HHS Secretary’s Advisory Committee on Human Research Protections (SACHRP) Guidance Convention on Human Rights and Biomedicine (Oveido Convention) 2017 Consensus Study Report of the National Academies of Sciences, Engineering and Medicine (NASEM), “Returning Individual Research Results to Participants: Guidance for a New Research Paradigm.” The NASEM framework emphasizes that *“[a]s the potential value of the result to participants and the feasibility of return increase, the justification for returning results becomes stronger.”*
**Justice considerations, including equitable DoC clinical trial access and accrual**	Despite an increase in DoC clinical trials over the past decade, access and enrollment remains highly irregular, especially in remote or underserved areas. Racial, ethnic and language-based disparities are also prevalent across a variety of clinical trial domains. Ethical considerations of justice and equity demand close attention to these potential disparities, and necessitate strategies to democratize access to DoC clinical trial enrollment and enhance representativeness.	NIH Revitalization Act of 1993, 21st Century Cures Act (2016) NIH Policy and Guidelines on the Inclusion of Women and Minorities as Subjects in Clinical Research (2017) FDA Guidance Document (2020) “Enhancing the Diversity of Clinical Trial Populations — Eligibility Criteria, Enrollment Practices, and Trial Designs Guidance for Industry” (FDA-2019-D-1264)
**Post-trial obligations and expanded access**	Researchers should consider obligations to participants and surrogates after trial concludes, along with approaches to expanded access/compassionate use of the investigational intervention in persons with DoC with no alternative treatment options.	FDA Guidance Document on Expanded Access to Investigational Drugs for Treatment Use (FDA-2013-D-0446, 2021) NIH Policy 3014-502 - Expanded Access, Including Emergency Use of Investigational Drugs, Biologics, and Medical Devices (Updated 2021) European Medicines Agency (EMA) Guideline on compassionate use of medicinal products, pursuant to Article 83 of Regulation (EC) No 726/2004 (2007)

## Data Availability

Not applicable.
